# Exercise-induced changes in skin temperature and blood parameters in horses

**DOI:** 10.5194/aab-62-205-2019

**Published:** 2019-04-16

**Authors:** Maria Soroko, Kinga Śpitalniak-Bajerska, Daniel Zaborski, Błażej Poźniak, Krzysztof Dudek, Iwona Janczarek

**Affiliations:** 1 Department of Horse Breeding and Equestrian Studies, Wroclaw University of Environmental and Life Sciences, Wrocław, 50-375, Poland; 2 Department of Environmental Hygiene and Animal Welfare, Wroclaw University of Environmental and Life Sciences, Wrocław, 50-375, Poland; 3 Department of Ruminants Science, West Pomeranian University of Technology, Szczecin, 71-270, Poland; 4 Department of Pharmacology and Toxicology, Wroclaw University of Environmental and Life Sciences, Wrocław, 50-375, Poland; 5 Faculty of Mechanical Engineering, Wroclaw University of Technology, Wrocław, 50-370, Poland; 6 Department of Horse Breeding and Use, University of Life Sciences in Lublin, Lublin, 20-950, Poland

## Abstract

The aim of the study was to assess the effects of training on haematological and biochemical blood parameters as well as on the changes in body surface temperature in horses. In order to identify the predictive value of surface temperature measurements as a marker of animal's performance, their correlations with blood parameters were investigated. The study was carried out on nine horses divided into two groups: routinely ridden and never ridden. Infrared thermography was used to assess surface temperature changes before (BT) and just after training (JAT) on a treadmill. Seven regions of interest (ROIs) located on the neck, shoulder, elbow, back, chest, gluteus and quarter were analysed. The blood samples were taken BT, JAT and 30 min after training (30AT). Haematological parameters including white blood cells, lymphocytes (LYMs), monocytes (MONOs), granulocytes (GRAs), eosinophils (EOSs), haematocrit (HCT) and platelets (PLTs) as well as biochemical parameters such as glucose (GLUC), urea, ${\mathrm{Na}}^{+}$, $K^{+}$ and ${\mathrm{Ca}}^{2+}$, and creatine phosphokinase (CPK) were analysed. Our results indicated a significant increase in surface temperature JAT (p=0.043) in the neck, shoulder, elbow, gluteus and quarter in routinely ridden horses. Significant changes in EOS (p=0.046) and HCT (p=0.043) in the case of the never-ridden and routinely ridden group, respectively, were found between the times of blood collection. In addition, there was a significant effect of the horse group and the time of blood collection on the CPK activity (p=0.025 to p=0.045) and urea concentrations (p=0.027 to p=0.045). In the routinely ridden horses, there were significant correlations between the changes in MONO (ρ=0.40), GRA (ρ=-0.40), PLT (ρ=-0.77), HCT (ρ=-0.36), GLUC (ρ=0.56) and urea (ρ=0.56) and the total ROI temperature changes. Moreover, significant correlations between the changes in MONO (ρ=-0.86), EOS (ρ=-0.65), GLUC (ρ=0.85), urea (ρ=0.85), ${\mathrm{Na}}^{+}$ (ρ=0.59) and $K^{+}$ (ρ=-0.85) and the total ROI temperature changes were found in never-ridden horses. Different changes in body surface temperature and blood parameters in routinely ridden and never-ridden horses could be associated with different conditioning and performance. A significantly higher surface temperature in routinely ridden horses, as well as the dynamics of changes in HCT, CPK and urea after training indicate better performance of these horses. Significant correlations between MONO, GLUC, and urea and a total ROI surface temperature as well as a negative correlation between MONO and the total ROI temperature in never-ridden horses indicated poor performance.

## 1 Introduction

A typical horse at rest produces approximately 50 kcal min${}^{-1}$ of heat by muscle resting metabolism, which is sufficient to maintain a proper internal body temperature (Mexiner, 1979). During exercise, approximately 70 %–80 % of the energy produced by working muscles is released as heat, and the amount of generated heat rapidly increases with work rate (Hodgson et al., 1994). The heat produced during exercise increases the temperature of the circulating blood (Hodgson et al., 1993) and subsequently increases the core temperature (Sexton et al., 1986). Blood flow through dermal capillaries increases, which leads to heat dissipation from the skin surface. Compared to humans, muscle tissue in horses makes up a much larger proportion of the total body mass (Hodgson et al., 1993). Therefore, heat produced by muscles during exercise in a horse must be lost to the environment very effectively in order to avoid hyperthermia.

Heat is dissipated from the skin surface to the surrounding environment mainly through convection, evaporation and infrared radiation (Guyton, 1991). Evaporation is due to the difference in the molecular pressure of water vapour between the skin surface and the environment, while the other components arise from the temperature difference between the skin surface and the environment. The exact contribution of these processes to heat loss during exercise is not fully understood. In horses, the balance of heat dissipation, transmission and storage may change in response to training time, gait type and speed as well as changes in the external environment (Hodgson et al., 1994).

Infrared thermography (IRT) is a non-invasive tool, which records the naturally emitted infrared radiation from the skin surface, providing a representation of body surface temperature distribution (Ring and Ammer, 2012; Soroko et al., 2017a). Previous studies used IRT to identify changes in skin surface temperature to monitor the underlying circulation, tissue metabolism and local blood flow in response to different environmental conditions (Mogg and Pollitt, 1992; Tunley and Henson, 2004; Soroko et al., 2015, 2017b). According to previous studies, treadmill exercise is predominantly an aerobic activity (Linder et al., 2003), and as such it leads to the increase in the blood flow to exercising muscles in order to meet the metabolic demands of the working tissues (Van de Graaffe et al., 1999). Simon et al. (2006) used IRT to highlight the influence of treadmill exercise in horses on body surface temperature changes. Similarly, Yarnell et al. (2014) assessed hindlimb's surface temperature changes associated with muscle contraction and alterations in associated blood flow in horses during treadmill exercises. The horses were exercised on a treadmill which ran dry and in two different levels of water height, which were the proximal interphalangeal joint and carpal joint. The increase in the water level caused greater effort and increased muscle activity detected by IRT. However, the increase in resistance (through the increase in water level) did not affect the occurrence of differences between temperatures in hindlimbs. Treadmill exercises have also been used to assess haematological and biochemical parameters in horses' performance (Kupczyński et al., 2018). Haematological and cardiovascular adaptations are necessary to guarantee the correct transport of oxygen and blood-borne substrates to muscles during exercise as well as to remove metabolites (Piccione et al., 2007). During physical exercises, one of the physiological changes is an increase in haematocrit (HCT), which has been recognized as a factor correlated with effort (Brun et al., 1990), similar to blood lactate and glucose levels (Piccione et al., 2007). The increase in the value of HCT is accompanied by an increase in the number of red blood cells (RBCs). The increase in these parameters facilitates the transport of oxygen and increases the buffer capacity of blood (Piccione et al., 2010). Training-induced anatomic adaptations like tissue remodelling are reflected by changes in plasma fibrinogen, urea, or levels of proteins and creatine phosphokinase (CPK) activity (Adamu et al., 2010). However, an increase in some blood biochemical parameters may be an important indicator of the overtraining syndrome. A 2- to 3-fold increase in CPK relative to its resting value may be indicative of muscle damage or injury (Bis-Wencel et al., 2011; Ostaszewski et al., 2012). According to Piccione et al. (2010), the interpretation of CPK activity should take into account the animal's clinical status, pathological symptoms and the stage of training.

The aim of the present study was to assess the effects of training on haematological and biochemical blood parameters as well as on the changes in body surface temperature in horses. In order to identify the predictive value of surface temperature measurements as a marker of an animal's performance, correlations with blood parameters were investigated.

## 2 Material and methods

### 2.1 Study animals

The study was carried out on a group of nine clinically healthy Felin ponies, aged between 2 and 17 years and with a body mass between 100 and 450 kg. The horses were divided into two groups according to a specific characteristic: group A comprised five routinely ridden horses and group B included four never-ridden horses. The animals in the two groups were subjected to slightly different exercise protocols, as detailed in Sect. 2.2, to account for anticipated differences in tolerance of exercise stress in routinely ridden and never-ridden animals. According to Adamu et al. (2014) and Soroko et al. (2017b), a different level of training causes different blood results and has an impact on body surface temperature differences. The study was approved by the Second Local Ethics Review Committee for Animal Experiments at the University of Life Sciences in Lublin, Poland, and performed at the Faculty of Veterinary Medicine once, over 1 d from 08:00 to 13:00 LT. The horses were fed with the same amount of hay 2 h prior to examination. They had ad libitum access to water throughout the experiment.

### 2.2 Treadmill exercise and dynamic infrared thermography

**Figure 1:**
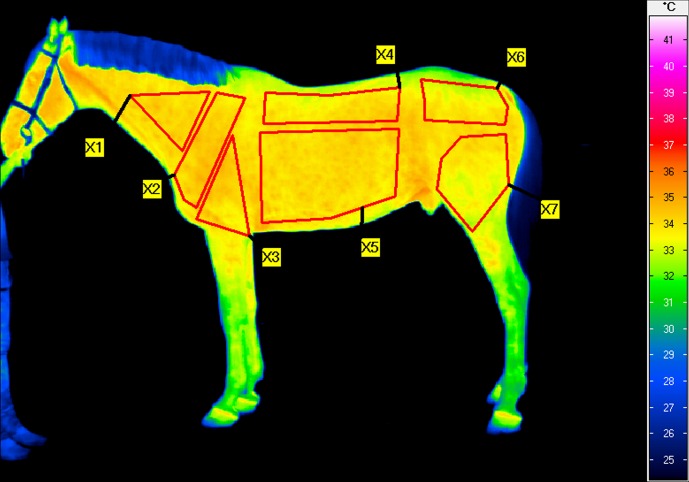
Example thermographic image of the left side of the horse before exercises on the treadmill with the seven regions of interest (ROIs) shown: neck (RO1), shoulder (ROI2), elbow (ROI3), back (ROI4), chest (ROI5), gluteus (ROI6), quarter (ROI7).

Thermographic images of the horses were taken using an InfraTec^®^ VarioCam HD resolution infrared camera (uncooled microbolometer focal plane array, focal plane array sensor size of 640×480, spectral range 7.5–14 µm, noise-equivalent temperature difference of <20 mK at 30 ${}^{\circ }$C, using the normal lens with IFOV (instantaneous field of view) of 0.57 mrad, measurement uncertainty of ±1 % of the overall temperature range; InfraTec Dresden, Germany). The horses were prepared for the study according to the established standards of thermographic examination in veterinary medicine (Purohit, 2009; Soroko and Howell, 2018). The protocol for treadmill exercises and thermographic assessment was similar to that described by Soroko et al. (2018). The horses were examined at rest before the daily exercise and were cleaned 1 h before the treadmill session. The manes and tails were plaited to ensure an unobstructed view of the neck and the hindlimb region. Each horse was individually taken from the stable to the building with the treadmill. Mean ambient temperature in the stable as well as in the examination facility was maintained at the level of 23 ${}^{\circ }$C with humidity 45 % (without major fluctuations). To minimize the influence of external environmental conditions, the doors of the examination room remained closed during both the acclimatization period for 1 h and the treadmill exercises (Turner, 2001). The ambient temperature in the examination room was measured by a TES 1314 thermometer (TES, Taipei, Taiwan). The horses were exercised on a Fizjo Pet treadmill (Hycon, Gdánsk, Poland) with their own head collar and with the lead rope held by the same familiar handler. Group A was exercised on the treadmill for 25 min, starting with walk (10 min), followed by 10 min of trot and then a further 5 min of walk, and the procedure ended with 10 min of recovery time (the horse standing at rest on the treadmill). Horses from group B were exercised on the treadmill for 20 min, starting with walk (10 min), followed by 5 min of trot and then 5 further minutes of walk, and the procedure ended with 10 min of recovery time. To standardize the exercise protocol, the treadmill acceleration, speed and exercise duration were computer-controlled. The speed of the treadmill varied depending on the individual horse. Each horse was assigned a comfortable active walking and trotting speed, with the mean walking speed for all horses being 3.86 km h${}^{-1}$ (standard deviation, SD, 0.67) and the mean trotting speed for all horses being 9.12 km h${}^{-1}$ (SD 0.82). The exercises were performed at relatively low work rates to avoid heat dissipation by evaporation of perspiration, which interferes with the measurement by reducing the horse's body surface temperature. Thermographic images of the left side of the horses before and just after the exercise were taken. The left side of the body was imaged at a 90${}^{\circ }$ camera angle from a distance of approximately 2 m from the horse. The thermographic images were analysed using IRBIS 3 Professional software (InfraTec, Dresden, Germany). In each thermographic image, seven regions of interest (ROIs) were defined on the basis of major muscle groups and bony landmarks visible on the thermograms (Fig. 1):

- the neck (ROI1), encompassing the serratus ventralis cervicis muscle;
- the shoulder (ROI2), encompassing the infraspinatus and supraspinatus muscles;
- the elbow (ROI3), encompassing the triceps brachii muscle;
- the back (ROI4), encompassing the longissimus muscle;
- the chest (ROI5), encompassing the chest muscles;
- the gluteus (ROI6), encompassing the gluteal muscles;
- the quarter (ROI7), encompassing the quarter muscle.

From each ROI, mean temperature (M) and SD were calculated.

### 2.3 Blood samples

Blood samples were taken three times from each horse: before training (BT), just after training (JAT) and after a 30 min post-training recovery period (30AT). Blood was taken from the external jugular vein (vena jugularis externa) using sterile Sarstedt tubes for serum (10 mL) and tubes with anticoagulant – EDTA (2 mL) (Sarstedt, Poland). Whole-blood hematological analyses were performed using ABC Vet analyzer (Horiba ABX, France). The following parameters were assessed: white blood cells (WBCs, K µL${}^{-1}$), lymphocytes (LYMs, K µL${}^{-1}$, %), monocytes (MONOs, K µL${}^{-1}$, %), granulocytes (GRAs, K µL${}^{-1}$, %), eosinophils (EOSs, K µL${}^{-1}$, %), platelets (PLTs, K µL${}^{-1}$) and haematocrit (HCT, %). In the blood serum, the following biochemical parameters were estimated: creatine phosphokinase activity (CPK, U L${}^{-1}$, where U is the enzyme unit, via the kinetic method, HORIBA ABX), glucose (GLUC, mg dL${}^{-1}$, via the oxidase method, HORIBA ABX reagents), urea (mg dL${}^{-1}$), sodium (${\mathrm{Na}}^{+}$, mmol L${}^{-1}$), potassium ($K^{+}$, mmol L${}^{-1}$) using an ion-selective adapter and calcium (${\mathrm{Ca}}^{2+}$, mmol L${}^{-1}$) via the colorimetric method (HORIBA ABX). The blood biochemistry analyses were carried out using a Pentra 400 biochemical analyser according to manufacturer's instructions (Horiba ABX, France).

### 2.4 Statistical analysis

Due to the lack of a normal distribution of the measured temperatures, blood haematological and biochemical parameters (verified with the Shapiro–Wilk W test), the results are presented as medians and ranges (max, min) in the tables. The significance of the differences in the ROI temperatures between group A and B was verified with the Mann–Whitney U test, and BT and JAT were calculated with the Wilcoxon matched pairs test. The significance of the differences in blood parameters at three time points – BT, JAT and 30AT – within each group was tested with the Friedman ANOVA and the Dunn post hoc test. The significance of the differences in blood parameters between group A and B (at each time point) was again verified with the Mann–Whitney U test.

To analyse the strength and direction of the relationships between the measured temperatures and blood parameters, the Spearman rank correlation coefficient (ρ) was calculated. Due to the lack of significant differences in the temperature increase caused by the training ($\Delta T=T_{\mathrm{JAT}}-T_{\mathrm{BT}}$) among the seven ROIs, the Spearman rank correlation coefficients between the mean temperature change in total ROI ($\Delta T_{\mathrm{total}}$) and the changes in blood parameters JAT were determined. The study did not include correlation between blood parameters 30AT and ROI surface temperature JAT as skin temperature would significantly change 30 min after exercise. In all the tests, statistical significance was reported when p≤0.05. All the calculations were performed using Statistica software (v. 12, StatSoft Inc., Tulsa, OK, USA).

## 3 Results

The descriptive statistics (medians and ranges) for the ROI temperatures as well as the haematological and biochemical blood parameters are presented in Tables 1 and 2, respectively. Surface temperature JAT (p=0.043) increased in group A for ROI1, ROI2, ROI3, ROI6 and ROI7 (Table 1).

**Table 1:** Median (min, max) of the temperature measurements in the selected regions of the body surface (ROIs) before the training on the treadmill (BT) and just after the training (JAT) in two groups of horses and the results of the non-parametric significance tests.

Body region	Group	Time		
		BT	JAT	p value${}^{*}$
ROI1	A	32.3 (31.9, 32.6)	33.4 (32.5, 34.2)	**0.043**
Neck (${}^{\circ }$C)	B	32.2 (30.4, 32.4)	32.4 (31.7, 33.6)	0.144
	p value${}^{**}$	0.221	0.221	–
ROI2	A	32.2 (31.3, 32.9)	33.5 (32.5, 34.0)	**0.043**
Shoulder (${}^{\circ }$C)	B	32.8 (30.4, 32.9)	32.3 (31.8, 34.4)	0.465
	p value${}^{**}$	0.389	0.221	–
RO3	A	31.7 (31.5, 32.7)	33.3 (32.6, 33.6)	**0.043**
Elbow (${}^{\circ }$C)	B	32.4 (30.3, 33.6)	32.2 (31.8, 35.4)	0.465
	p value${}^{**}$	0.806	0.327	–
ROI4	A	31.2 (30.1, 33.0)	33.0 (31.9, 33.8)	0.080
Back (${}^{\circ }$C)	B	31.4 (30.5, 32.5)	32.2 (31.7, 33.7)	0.144
	p value${}^{**}$	0.806	0.980	–
ROI5	A	32.0 (30.8, 33.2)	33.2 (32.0, 34.0)	0.080
Chest (${}^{\circ }$C)	B	32.2 (30.7, 32.9)	32.4 (31.7, 34.6)	0.273
	p value${}^{**}$	0.806	0.806	–
ROI6	A	31.2 (30.4, 32.4)	33.0 (31.7, 33.5)	**0.043**
Gluteus (${}^{\circ }$C)	B	31.1 (30.0, 31.5)	31.9 (31.6, 33.5)	0.068
	p value${}^{**}$	0.806	0.462	–
ROI7	A	31.6 (30.4, 32.8)	33.7 (32.1, 34.0)	**0.043**
Quarter (${}^{\circ }$C)	B	32.0 (30.7, 32.8)	32.6 (32.0, 34.8)	0.144
	p value${}^{**}$	1.000	0.462	–

${}^{*}$ Wilcoxon matched pairs test; ${}^{**}$ Mann–Whitney U test; A – routinely ridden horses (n=5); B – never-ridden horses (n=4); bold values denote statistical significance at p<0.05.

**Table 2:** Median (min, max) blood test results before the training on the treadmill (BT), just after the training (JAT) and 30 min after the training (30AT) and the results of the non-parametric significance tests.

Parameter	Group	Time			p value${}^{*}$
		BT	JAT	30AT	
WBC (K µL${}^{-1}$)	A	6.0 (5.4, 9.4)	6.9 (5.5, 10.2)	6.2 (5.3, 9.2)	0.247
	B	6.7 (6.5, 9.4)	7.6 (6.1, 9.9)	6.8 (6.2, 8.5)	0.472
	p value${}^{**}$	0.385	0.806	0.539	–
LYM (K µL${}^{-1}$)	A	1.7 (1.1, 2.7)	2.1 (1.4, 2.4)	1.7 (1.2, 1.9)	0.091
	B	2.5 (1.6, 2.9)	2.9 (1.5, 3.6)	2.6 (1.4, 3.1)	0.472
	p value${}^{**}$	0.217	0.213	0.142	–
MONO (K µL${}^{-1}$)	A	0.1 (0.1, 0.3)	0.2 (0.1, 0.4)	0.2 (0.1, 0.3)	0.247
	B	0.3 (0.1, 0.3)	0.2 (0.2, 0.3)	0.2 (0.1, 0.3)	0.761
	p value${}^{**}$	0.295	0.662	0.793	–
GRA (K µL${}^{-1}$)	A	4.6 (3.7, 7.4)	5.0 (3.7, 7.4)	4.5 (3.2, 7.1)	0.211
	B	4.5 (3.8, 6.2)	4.5 (4.3, 6.3)	4.3 (3.6, 5.5)	0.174
	p value${}^{**}$	0.902	1.000	0.624	–
EOS (K µL${}^{-1}$)	A	0.13 (0.12, 0.35)	0.12 (0.08, 0.28)	0.11 (0.08, 0.23)	0.051
	B	0.24 (0.19, 0.28)	0.26 (0.13, 0.30)	0.18 (0.11, 0.23)	**0.039**
	p value${}^{**}$	0.140	0.085	0.264	–
PLT (K µL${}^{-1}$)	A	190 (144, 235)	197 (146, 218)	205 (168, 247)	0.854
	B	201 (119, 248)	222 (194, 243)	217 (197, 220)	0.472
	p value${}^{**}$	1.000	0.142	0.462	–
HCT (%)	A	32.2 (30.4, 41.1)	39.2 (31.5, 44.1)	31.3 (27.0, 37.5)	**0.015**
	B	34.0 (29.9, 37.7)	33.3 (31.1, 35.6)	31.4 (28.4, 32.2)	0.174
	p value${}^{**}$	0.806	0.142	0.624	–
CPK (U L${}^{-1}$)	A	155 (143, 158)	181 (176, 229)	370 (267, 413)	**0.007**
	B	210 (157, 217)	212 (168, 270)	236 (204, 273)	0.472
	p value${}^{**}$	**0.037**	0.711	**0.027**	–
GLUC (mg dL${}^{-1}$)	A	97 (91, 109)	92 (87, 97)	111 (92, 121)	0.247
	B	96 (95, 97)	98 (89, 101)	102 (91, 109)	0.779
	p value${}^{**}$	0.806	0.327	0.327	–
Urea (mg dL${}^{-1}$)	A	32.8 (29.7, 35.6)	31.8 (27.1, 34.2)	30.6 (29.1, 32.5)	0.091
	B	29.4 (28.6, 29.7)	44.2 (30.3, 45.9)	31.7 (28.2, 32.3)	**0.039**
	p value${}^{**}$	**0.027**	0.086	0.806	–
${\mathrm{Na}}^{+}$ (mmol L${}^{-1}$)	A	139 (138, 141)	135 (134, 139)	136 (135, 138)	0.076
	B	139 (138, 140)	141 (134, 142)	136 (134, 137)	0.074
	p value${}^{**}$	0.899	0.174	0.530	–
$K^{+}$ (mmol L${}^{-1}$)	A	3.2 (2.8, 3.8)	4.3 (3.6, 4.5)	3.0 (2.3, 4.4)	0.066
	B	3.4 (2.3, 4.2)	3.1(2.1, 3.8)	3.7 (3.2, 3.9)	0.607
	p value${}^{**}$	0.902	**0.027**	0.624	–
${\mathrm{Ca}}^{2+}$ (mmol L${}^{-1}$)	A	1.65 (1.63, 1.77)	1.73 (1.60, 1.84)	1.59 (1.52, 1.66)	0.091
	B	1.73 (1.69, 1.78)	1.63 (1.60, 1.83)	1.67 (1.52, 1.75)	0.174
	p value${}^{**}$	0.084	0.459	0.389	–

${}^{*}$ Friedman ANOVA; ${}^{**}$ Mann–Whitney U test; A – routinely ridden horses (n=5); B – never-ridden horses (n=4); WBCs – white blood cells; LYMs – lymphocytes; MONOs – monocytes; GRAs – granulocytes; EOSs – eosinophils; PLTs – platelets; HCT – haematocrit; CPK – creatine phosphokinase; GLUC – glucose; bold values denote statistical significance at p<0.05.

There were significant differences in EOS (p=0.046) in group B: BT (Me, median = 0.24 K µL${}^{-1}$), JAT (Me = 0.26 K µL${}^{-1}$) as well as 30AT (Me = 0.18 K µL${}^{-1}$). A similar difference in HCT (p=0.043) was found in group A: BT (Me = 32.2 %) and JAT (Me = 39.2 %) as well as 30AT (Me = 31.3 %) (Tables 2 and 3). The time of blood collection significantly affected the CPK activity in group A (Table 2), which was higher 30AT (Me = 370 U L${}^{-1}$) than BT (Me = 155 U L${}^{-1}$; p=0.025) and JAT (Me = 181 U L${}^{-1}$; p=0.045) (Table 3). In addition, the CPK activity JAT differed significantly from that BT (p=0.043). Significant differences in the CPK activity were also found between both groups at individual time points (BT – 155 U L${}^{-1}$ vs. 210 U L${}^{-1}$, p=0.037; 30AT – 370 U L${}^{-1}$ vs. 236 U L${}^{-1}$, p=0.027) (Table 2). A significant effect of the time of blood collection was also noticed for the plasma urea level in group B, which first increased (Me = 29.4 mg dL${}^{-1}$ BT and Me = 44.2 mg dL${}^{-1}$ JAT; p=0.045) and 30AT decreased almost to the initial value (Me = 31.7 mg dL${}^{-1}$; p=0.317). In addition, a significant difference was observed in the urea content JAT and 30AT (p=0.045). Moreover, the urea concentration was significantly higher in group A (Me = 32.8 mg dL${}^{-1}$) compared to group B (Me = 29.4 mg dL${}^{-1}$) BT (p=0.027). The $K^{+}$ level in blood differed significantly between group A (Me = 4.3 mmol L${}^{-`1}$) and B (Me = 3.1 mmol L${}^{-1}$) JAT (p=0.027). There was no difference in the values of other haematological and biochemical parameters among the time points from both groups. The Spearman rank correlation coefficients (ρ) between the changes in blood parameters and total ROI surface temperature measured BT and JAT are presented in Table 4. Significant correlations were found between the changes in MONO (ρ=0.40), GRA (ρ=-0.40), PLT (ρ=-0.77), HCT (ρ=-0.36), GLUC (ρ=0.56) and urea (ρ=0.56) and the total temperature changes in group A as well as between the changes in MONO (ρ=-0.86), EOS (ρ=-0.65), GLUC (ρ=0.85), urea (ρ=0.85), ${\mathrm{Na}}^{+}$ (ρ=0.59) and $K^{+}$ (ρ=-0.85) and the total temperature changes in group B.

**Table 3:** Multiple comparisons of the mean ranks for all groups (p values) before the training on the treadmill (BT), just after the training (JAT) and 30 min after the training (30AT).

	EOS (K µL${}^{-1}$) – group B				HCT (%) – group A				CPK (U L${}^{-1}$) – group A				urea (mg dL${}^{-1}$) – group B		
	BT	JAT	30AT		BT	JAT	30AT		BT	JAT	30AT		BT	JAT	30AT
BT	x	0.317	**0.046**		x	**0.043**	0.080		x	**0.043**	**0.025**		x	**0.045**	0.317
JAT		x	**0.046**			x	**0.043**			x	**0.045**			x	**0.045**
30AT			x				x				x				x

A – routinely ridden horses (n=5); B – never-ridden horses (n=4); EOSs – eosinophils; HCT – haematocrit; CPK – creatine phosphokinase; bold values denote statistical significance at p<0.05.

**Table 4:** Values of Spearman's rank correlation coefficients (ρ) between the changes in body surface temperature (ΔT) and blood parameters in the horses from two groups.

Change in the	$\Delta T_{\mathrm{total}}$ (${}^{\circ }$C)	
parameter value	group A	group B
(JAT – BT)		
ΔWBC (K µL${}^{-1}$)	-0.192	-0.115
ΔLYM (%)	0.244	0.091
ΔMONO (%)	**0.404**	-0.858
ΔGRA (%)	-0.404	-0.091
ΔEOS (%)	-0.212	-0.653
ΔPLT (K µL${}^{-1}$)	-0.771	-0.091
ΔHCT (%)	-0.358	-0.178
ΔCPK (U L${}^{-1}$)	0.120	-0.162
ΔGLUC (mg dL${}^{-1}$)	**0.563**	**0.850**
Δurea (mg dL${}^{-1}$)	**0.563**	**0.850**
Δ${\mathrm{Na}}^{+}$ (mmol L${}^{-1}$)	0.049	**0.589**
Δ$K^{+}$ (mmol L${}^{-1}$)	-0.326	-0.850
Δ${\mathrm{Ca}}^{2+}$ (mmol L${}^{-1}$)	-0.128	-0.312

WBCs – white blood cells; LYMs – lymphocytes; MONOs – monocytes; GRAs – granulocytes; EOSs – eosinophils; PLTs – platelets; HCT – haematocrit; CPK – creatine phosphokinase; GLUC – glucose; bold values denote statistical significance at p<0.05.

## 4 Discussion

In the current study, a significant training-induced increase in body surface temperature was observed in the neck, shoulder, elbow, gluteus and quarter regions only in routinely ridden horses. The increased surface temperature must have been associated with the longer time of training in trot on the treadmill compared to the never-ridden horses. That contributed to the increased heat production by muscle contractions and subsequent increase in the local blood circulation in order to meet the metabolic and thermoregulatory demands of the working tissues. During exercise, muscles, tendons, ligaments and bones are subject to overloads. This affects the rate of biochemical processes in individual tissues with the increased oxygen demand in muscles being the most eminent example. This leads to intensified muscle perfusion and eventually to increased skin perfusion and improved dissipation of excess heat to the environment (Hinchcliff et al., 2008). Earlier studies indicated a model for surface temperature distribution of forelimbs and hindlimbs before and after exercises (Jodkowska and Dudek, 2000; Jodkowska, 2005). It was indicated that surface temperature depended on the type of exercise and was considerably higher in forelimbs compared to hindlimbs after training (Jodkowska et al., 2001), whereas in our study, temperatures increased in both forelimbs (shoulder, elbow) and hindlimbs (gluteus and quarter). Previous studies reported that neck area was the warmest area of the horse body before and after training (Jodkowska and Dudek, 2000). Jodkowska et al. (2011) indicated that the highest temperatures after training were recorded in both neck and thigh areas. Similar results were observed in the current study, where neck, and quarter areas showed a significant temperature increase. One of the first studies on the influence of treadmill exercises on body surface temperature changes in the forelimbs and hindlimbs using IRT was published by Simon et al. (2006). In that study, surface temperature in upper parts of the forelimbs and hindlimbs after exercise increased by 6 ${}^{\circ }$C, whereas for the distal parts of the limbs surface temperature increased by 8 ${}^{\circ }$C. However, only limited parts of the body were investigated in that study.

The results of the current study indicated that the analysed blood constituents changed in response to work on the treadmill. Lower mean EOS level was found in the routinely ridden horses. In a study on endurance horses, Adamu et al. (2010) found that a lower EOS level was related to good performance. On the other hand, an excessive increase in GRA and EOS may be related to intense physical exertion and stress (Winnicka, 2004). In both groups, the increase in the surface temperature caused a decrease in the total EOS and this was significant in the case of never-ridden horses (ρ=-0.653). Also, MONO (ρ=-0.858; group B) significantly decreased only in the never-ridden horses. The rise in the ROI temperature (lower than that in group A) associated with the decreased EOS and MONO after training may be due to the weaker condition of the horses from group B or probably less efficient thermoregulation mechanisms associated with heat dissipation. The type of exercise could induce a rise in body temperature and immune disturbances (Niess et al., 2003), which can account for the negative correlation between the concentration of EOS and MONO and the total ROI surface temperature.

In this study, a significant correlation between the total ROI surface temperature and PLT in routinely ridden horses (ρ=-0.771) was found, which was probably associated with a wide range of individual variability within the group. Exercises, especially in young horses, activate the production of the factors responsible for clotting (Assenza et al., 2013). In the case of the routinely ridden horses, a greater increase in the concentration of HCT immediately after exercise (a consequence of spleen contraction) and quick return to the rest value (Piccione et al., 2010) indicated a better adaptation of the horses to physical effort. This also explains the significant correlation between HCT and the ROI temperature (ρ=-0.358).

A post-training increase in CPK activity was found in both groups; however, in the routinely ridden group this increase was statistically significantly higher and reached 370 U L${}^{-1}$ after 30 min rest. CPK activity is associated with intramuscular energy processes (Fazio et al., 2014). This enzyme's activity in animals' serum is characterized by high variability (Bis-Wencel et al., 2011). For instance, CPK activity at 255 U L${}^{-1}$ in polo ponies at rest is considered moderate (Ferraz et al., 2010). However, Ostaszewski et al. (2012) reported that CPK activity over 200 U L${}^{-1}$ may indicate muscle damage. Winnicka (2004) reported a wide reference range for CPK activity in healthy horses from 90 to 565 U L${}^{-1}$. In this study, there was a numeric increase in the GLUC level after 30 min post-training rest; however, it was not statistically significant. The increase in GLUC concentration after training in healthy horses is proportional to the intensity of effort and the level of lactate in blood. Resting values of the blood GLUC in horses usually vary from 5.0 to 6.5 mg dL${}^{-1}$ (Bis-Wencel et al., 2011), which corresponds to the values obtained in the present study. Post-exertional rise in blood GLUC concentration can be a consequence of the increase in energy mobilization from fat tissue (Fazio et al., 2014). In the case of performance horses, GLUC concentration is usually lower, which may indicate a more economical management of glucose homeostasis and the use of free fatty acid as an energy source (Adamu et al., 2014). During a short-term effort, the main energy source is glucose; therefore, in the early stage of physical effort, an initial decrease followed by an increase in blood glucose is indicated (Bis-Wenel et al., 2011). A similar pattern (although not significant) was observed in the routinely ridden horses from the present study. The increase in ROI temperature had a significant effect on the increase in the blood glucose levels in both groups (ρ=0.563 and ρ=0.850). This may have resulted from the increase in energy demands for thermoregulatory mechanisms removing excess heat from the body during exercise.

The resting values of urea in the routinely ridden horses were higher compared to the never-ridden group. However, in the case of the never-ridden group, the blood urea increase was high just after the training session, which could be related to the weaker condition of these horses, but these changes were within the reference range (Winnicka, 2004). The increased serum urea concentration may be attributed to the massive fluid loss by sweating and subsequent (transient) reduction in the renal blood flow. Intensive training of athletic horses can lead to kidney dysfunction as indicated by a high level of serum urea (Piccione et al., 2010). In addition, a significant correlation between urea and the total ROI temperature (ρ=0.563 and ρ=0.850 for group A and B, respectively) was observed, and this also could be associated with thermoregulatory mechanisms by sweating.

A post-exertional decrease in the serum ${\mathrm{Na}}^{+}$ concentration has been demonstrated in most of the studies conducted so far in horses (Muñoz et al., 2008). Increased losses of ${\mathrm{Na}}^{+}$ or $K^{+}$ are usually observed in horses with a lower level of performance (Aguilera-Tejero et al., 2000) and can be related to the increased temperature and massive fluid loss due to sweating; however, the concentration of ${\mathrm{Na}}^{+}$ increased with the rise in the ROI temperature in the never-ridden horses, which was significantly high (ρ=0.59). In the present study, it was found that the increase in the body surface temperature just after training can indicate the decrease in the serum $K^{+}$ concentration in the never-ridden horses. It is important to note that $K^{+}$, similarly to ${\mathrm{Na}}^{+}$, is essential for the proper functioning of muscles and neural tissues. During exercise, a large amount of heat is produced in the body, which has to be dissipated in order to maintain thermostasis. The significant correlation (ρ=-0.850) between $K^{+}$ concentration and body surface temperature may result from the stimulation of thermoregulatory mechanisms to remove excess heat from the body. One of the mechanisms is the change in blood volume which may also affect serum $K^{+}$ concentration. On the other hand, dehydration can inhibit the body's ability to remove the excess heat which can affect the thermoregulatory mechanism (Sawka et al., 2001). Therefore, the IRT examination in relation to the level of electrolytes in the blood might be helpful in diagnosing heat stress. This further supports the tight connection of blood $K^{+}$ and ${\mathrm{Na}}^{+}$ levels with thermoregulatory processes in horses.

## 5 Conclusions

Different changes in body surface temperature and blood parameters in routinely ridden and never-ridden horses could be associated with their different conditioning and performance. Our results indicate the significantly higher ROI surface temperature in routinely ridden horses (neck, back, gluteus and quarter) as well as the dynamics of changes after training in HCT, CPK and urea, which may indicate a better performance of these horses. Significant correlations between MONO, GLUC and urea and a total ROI surface temperature and a negative correlation between MONO and the total ROI in never-ridden horses indicated poor performance.

## Data Availability

The data used in the present study are confidential and therefore are not publicly accessible.
